# Neighbourhood socioeconomic position, prenatal care and fulfilment of postpartum permanent contraception: Findings from a multisite cohort study

**DOI:** 10.1002/rfc2.64

**Published:** 2023-10-30

**Authors:** Kristen A. Berg, Brooke W. Bullington, Douglas D. Gunzler, Emily S. Miller, Margaret Boozer, Tania Serna, Jennifer L. Bailit, Kavita S. Arora

**Affiliations:** 1 Center for Health Care Research and Policy, Population Health Research Institute, MetroHealth Medical System, Cleveland, Ohio, USA; 2 School of Medicine, Case Western Reserve University, Cleveland, Ohio, USA; 3 Department of Epidemiology, Gillings School of Global Public Health, University of North Carolina at Chapel Hill, Chapel Hill, North Carolina, USA; 4 Carolina Population Center, University of North Carolina at Chapel Hill, Chapel Hill, North Carolina, USA; 5 Department of Obstetrics and Gynecology, Division of Maternal Fetal Medicine, Warren Alpert Medical School of Brown University, Providence, Rhode Island, USA; 6 Department of Obstetrics and Gynecology, University of Alabama at Birmingham, Birmingham, Alabama, USA; 7 Department of Obstetrics and Gynecology, University of California San Francisco, San Francisco, California, USA; 8 Department of Obstetrics and Gynecology, MetroHealth Medical System, Cleveland, Ohio, USA; 9 Department of Obstetrics and Gynecology, University of North Carolina, Chapel Hill, North Carolina, USA

**Keywords:** ADI, ADI-3, neighbourhood socioeconomic disadvantage, prenatal care, reproductive health equity, sterilization

## Abstract

**Introduction:**

Research suggests neighbourhood socioeconomic vulnerability is negatively associated with women’s likelihood of receiving adequate prenatal care and achieving desired postpartum permanent contraception. Receiving adequate prenatal care is linked to a greater likelihood of achieving desired permanent contraception, and access to such care may be critical for women with Medicaid insurance given that the federally mandated Medicaid sterilization consent form must be signed at least 30 days before the procedure. We examined whether adequacy of prenatal care mediates the relationship between neighbourhood socioeconomic position and postpartum permanent contraception fulfilment, and examined moderation of relationships by insurance type.

**Methods:**

This secondary analysis of a retrospective cohort study examined 3012 Medicaid or privately insured individuals whose contraceptive plan at postpartum discharge was permanent contraception. Path analysis estimated relationships between neighbourhood socioeconomic position (economic hardship and inequality, financial strength and educational attainment) and permanent contraception fulfilment by hospital discharge, directly and indirectly through adequacy of prenatal care. Multigroup testing examined moderation by insurance type.

**Results:**

After adjusting for age, parity, weeks of gestation at delivery, mode of delivery, race, ethnicity, marital status and body mass index, having adequate prenatal care predicted achieving desired sterilization at discharge (*β* = 0.065, 95% confidence interval [CI]: 0.011, 0.117). Living in neighbourhoods with less economic hardship (indirect effect −0.007, 95% CI: −0.015, −0.001), less financial strength (indirect effect −0.016, 95% CI: −0.030, −0.002) and greater educational attainment (indirect effect 0.012, 95% CI: 0.002, 0.023) predicted adequate prenatal care, in turn predicting achievement of permanent contraception by discharge. Insurance status conditioned some of these relationships.

**Conclusion:**

Contact with the healthcare system via prenatal care may be a mechanism by which neighbourhood socioeconomic disadvantage affects permanent contraception fulfilment, particularly for patients with Medicaid. To promote reproductive autonomy and healthcare equity, future inquiry and policy might closely examine how neighbourhood social and economic characteristics interact with Medicaid mandates.

## INTRODUCTION

Permanent contraception (i.e., sterilization) is a highly effective form of contraception, and is commonly used by women in the United States.^1^ However, lower-income and racially and ethnically minoritized women experience a lower likelihood of achieving postpartum permanent contraception compared to White and higher-income women.^2^ Reasons for this disparity include patient-related factors as well as barriers at the physician, hospital and policy levels. One plausible reason for this disparity is the federal Medicaid sterilization policy, which requires a 30-day waiting period before sterilization for women with Medicaid insurance.^3,4^ Reports suggest that over half of permanent contraception procedures are done during the immediate postpartum period,^5^ where some of these barriers to care are minimized (e.g., accessibility of insurance coverage,^6^ scheduling convenience for the patient, and technical ease for the physician^7^). In order for a postpartum permanent contraception procedure to occur before hospital discharge for a patient with Medicaid insurance, the federally required consent form must be signed during prenatal care, at least 30 days in advance, thus requiring access to prenatal care.

A small body of research suggests that neighbourhood conditions may be related to the use of permanent contraception; for example, neighbourhoods with greater household income have demonstrated lower proportions of permanent contraception use among women.^8^ However, research employing more robust area-based measures of socioeconomic position finds that living in more a resource-vulnerable neighbourhood is associated with lower odds of achieving desired postpartum permanent contraception,^9,10^ and that such effects are exacerbated among patients with Medicaid.^10^ Given the importance of healthcare system access for obtaining prenatal care, and that characteristics of the neighbourhood environment are associated with healthcare access and utilization,^11^ one mechanism linking the neighbourhood with the achievement of permanent contraception may be the adequacy of prenatal care. Receiving adequate prenatal care has been shown to increase women’s likelihood of achieving desired permanent contraception,^12,13^ but living in more socioeconomically vulnerable neighbourhoods may negatively affect women’s chances of obtaining adequate prenatal care.^14^ Prior work has found that greater neighbourhood economic distress is associated with late entry to prenatal care^15^ and that other neighbourhood characteristics, such as higher proportions of unemployment or single-headed households, are associated with inadequate prenatal care.^16^

Limited research exists linking neighbourhood socioeconomic position and permanent contraception, and most existing studies are single-site.^10^ Furthermore, studies have scarcely explored possible mechanisms underpinning relationships between the neighbourhood environment and the fulfilment of permanent contraception. Methodologically, other studies have been constrained by the use of measures assessing community vulnerability to climate disaster rather than those that are empirically valid measures of socioeconomic position,^9^ and are not specific about potential mechanisms of neighbourhood socioeconomic position that may affect fulfilment of permanent contraception. Thus, we aimed to examine, in a multiinstitution cohort, the direct and indirect effects of multidimensionally measured neighbourhood socioeconomic position on the fulfilment of postpartum permanent contraception as mediated by the adequacy of prenatal care. Given Medicaid policies affecting permanent contraception fulfilment, we additionally examined whether such associations would vary according to insurance type. We hypothesized that (1) living in more socioeconomically vulnerable neighbourhoods would be negatively associated with adequacy of prenatal care which, in turn, would predict a lower likelihood of achieving permanent contraception; and (2) that such relationships would differ for women with Medicaid compared to private insurance.

## MATERIALS AND METHODS

### Sample

Data originate from a retrospective cohort of patients who delivered between 1 January 2018 and 31 December 2019, across four US hospitals: MetroHealth Medical System in Ohio, Northwestern Memorial Hospital in Illinois, the University of Alabama at Birmingham in Alabama and the University of California San Francisco in California. Women were included in the study who (1) had a contraception plan of permanent contraception as documented in their discharge summary or last inpatient progress note and (2) delivered at 20 or more weeks’ gestation. Exclusion criteria included (1) prior fulfilment of permanent contraception with conception via in vitro fertilization; (2) undergoing caesarean hysterectomy due to suspected placenta accrete and (3) peripartum mortality. For multiple deliveries within the study period wherein permanent contraception was desired, only the earliest delivery was retained for analysis. Clinical and demographic data such as insurance type, timing and status of permanent contraception fulfilment and co-occurring medical conditions were abstracted from the electronic medical record. Full detail on study methods and procedures has been published previously.^13^ The study was approved by the MetroHealth Medical System institutional review board.

### Outcomes

Our focal outcome variable was the fulfilment of permanent contraception before hospital discharge (binary). Our mediating variable was the adequacy of prenatal care as operationalized by the Kotelchuck Index^17^ (binary; adequate or inadequate) which integrates two critical elements of, first, when prenatal care began and, second, the number of prenatal care visits relative to when care began.

### Focal predictors

Our focal exposures in this analysis were three domains of neighbourhood socioeconomic position as operationalized by the Area Deprivation Index-3^18^ (ADI-3). Defined by factor analytic methods, the ADI-3 is a construct-validated, revised version of Singh’s^19^ original ADI. The ADI-3 measures three empirically distinct but related latent subdimensions of neighbourhood socioeconomic position: neighbourhood economic hardship and inequality (hereafter, ‘economic hardship’) (e.g., the ratio of households earning income less than $10 000 to those earning $50 000 or more; percent of households without a motor vehicle), financial strength (e.g., median family income in dollars; median home value in dollars) and educational attainment (e.g., percent of the population aged 25 years or older with at least a high school diploma). We assembled ADI-3 scores using the open-source ‘sociome’^20^ and ‘tidycensus’^21^ packages in R version 4.4.4.^22^ Both packages request place- and time-bound indicators from the US Census Application Programming Interface, and data transformations are conducted by the software according to published specifications.^21^ In the current study, we used 2019 5-year American Community Survey estimates with the United States as the reference geography. Raw ADI and ADI-3 scores are standardized, within ‘sociome’, to have a mean value of 100 and standard deviation of 20 and are scored such that greater scores indicate greater values of the named ADI-3 subdomain (e.g., a higher score on ‘economic hardship’ indicates that a neighbourhood is characterized by greater economic hardship, and a higher score on ‘educational attainment’ indicates that a neighbourhood is characterized by higher levels of educational attainment). We used ArcGIS version 10.6.1 to geocode patients’ home address, at the time of delivery, to their census tract.

### Covariates

Covariates included insurance type (binary; Medicaid or private) as abstracted from billing records, self-reported race (categorical, dummy-coded with White as a reference; Black, Asian, other and declined or unknown) and ethnicity (binary; Hispanic or non-Hispanic); marital status (binary; married or not married), maternal age at delivery (continuous in years), parity (i.e., number of pregnancies reaching at least 20 weeks) up until delivery (binary; less than two children, two or more), weeks of gestation at delivery (continuous), delivery type (binary; Caesarean or vaginal) and body mass index (BMI; continuous) during pregnancy. Covariates were selected given that they have been measured to vary with women’s contraception decision-making, including their desire for permanent contraception.^13^

### Analytic approach

We descriptively analyzed the sample’s demographic and clinical characteristics stratified by fulfilment of permanent contraception and adequacy of prenatal care. Following, multivariate analyses proceeded in two stages. First, we estimated a path analysis to model the effects of neighbourhood economic hardship, financial strength and educational attainment on the fulfilment of permanent contraception. This included both direct effects as well as indirect (mediation) effects through the adequacy of prenatal care. Both mediator and outcome variables were regressed on all covariates. Path analysis is a special case of structural equation modelling (SEM) and is a powerful multivariate technique that merges directional, hypothesis-driven conceptual models and a system of linked, regression-like equations to measure complex relationships among observed variables wherein some variables (e.g., adequacy of prenatal care) may function simultaneously as both dependent and independent variables.^23,24^

For analytical purposes in modelling binary outcome and mediator variables using an SEM framework, it is assumed that a normally distributed latent variable exists from which each observed categorical level (e.g., the two levels of adequacy of prenatal care and achievement of permanent contraception) is derived when the latent variable exceeds some threshold value (i.e., the value of the latent variable at which a patient transitions from a value of zero, or ‘no’, on whether they are predicted to have adequate prenatal or to have achieved permanent contraception, to a value of one or ‘yes’).^24,25^ Adequacy of prenatal care* and postpartum permanent contraception fulfilment* are latent variables, each with one threshold. For the mediator variable measuring the adequacy of prenatal care, the value is zero, or ‘no’/’not having adequate prenatal care’ if the value of adequacy of prenatal care* is less than the threshold, and the value is one, or ‘yes’/’having adequate prenatal care’ if the value of adequacy of prenatal care* is greater than the threshold. Similarly, for the outcome variable measuring the achievement of permanent contraception, the value is zero, or ‘no’/’did not achieve postpartum permanent contraception’ if the value of postpartum permanent contraception fulfilment* is less than the threshold, and the value is one, or ‘yes’ if the value of postpartum permanent contraception fulfilment* is greater than the threshold.

As such, interpretations of path model coefficients follow probit estimates where coefficients designate the change in cumulative normal probability of the outcome variable given a unit increase in the predictor.^26^ Specifically, a coefficient refers to a unit increase in the *z* score (number of standard deviations from the mean) for the probability of being in the ‘having adequate prenatal care’ or ‘did achieve postpartum permanent contraception’ categories versus the reference categories of not having adequate prenatal care or not achieving permanent contraception.^24^ For example, in our model, a path coefficient of 0.20 between the predictor neighbourhood educational attainment and the mediator adequacy of prenatal care would suggest that with each point increase in neighbourhood educational attainment, the *z* score in adequacy of prenatal care is 0.2 standard deviations above the mean in the continuous latent response of adequacy of prenatal care*.

Second, after estimating the aforementioned path analysis, we examined moderation by insurance status by employing invariance (i.e., multigroup) testing techniques to examine where and how model parameters varied according to women’s insurance. We referred to the following generally recommended indices and thresholds to discern models’ goodness of fit: comparative fit index (CFI) values of at least 0.90 indicated adequate fit, and root mean square error of approximation (RMSEA) and standardized root-mean-square residual (SRMR) values less than 0.05 indicated good fit.^27–29^ For assessing model comparison, CFI and RMSEA changes greater than |0.01| and |0.03| were taken as evidence of nonequivalence and sufficient reason to reject the constrained model.^24,30,31^ Model modification indices were inspected during model comparisons to highlight specific areas of model misfit and thus direct the release of equivalence constraints for individual model parameters.^32^

All multivariate modelling was performed in Mplus version 8.3 using theta parameterization and the weighted least square mean and variance adjusted estimator to accommodate binary outcome and mediator variables. Bootstrap sampling was employed to test for indirect effects^33^ and estimate standard errors robust to assumptions such as multivariate normality,^34^ and standardized effects are presented.

## RESULTS

### Descriptive results

**FIGURE 1:**
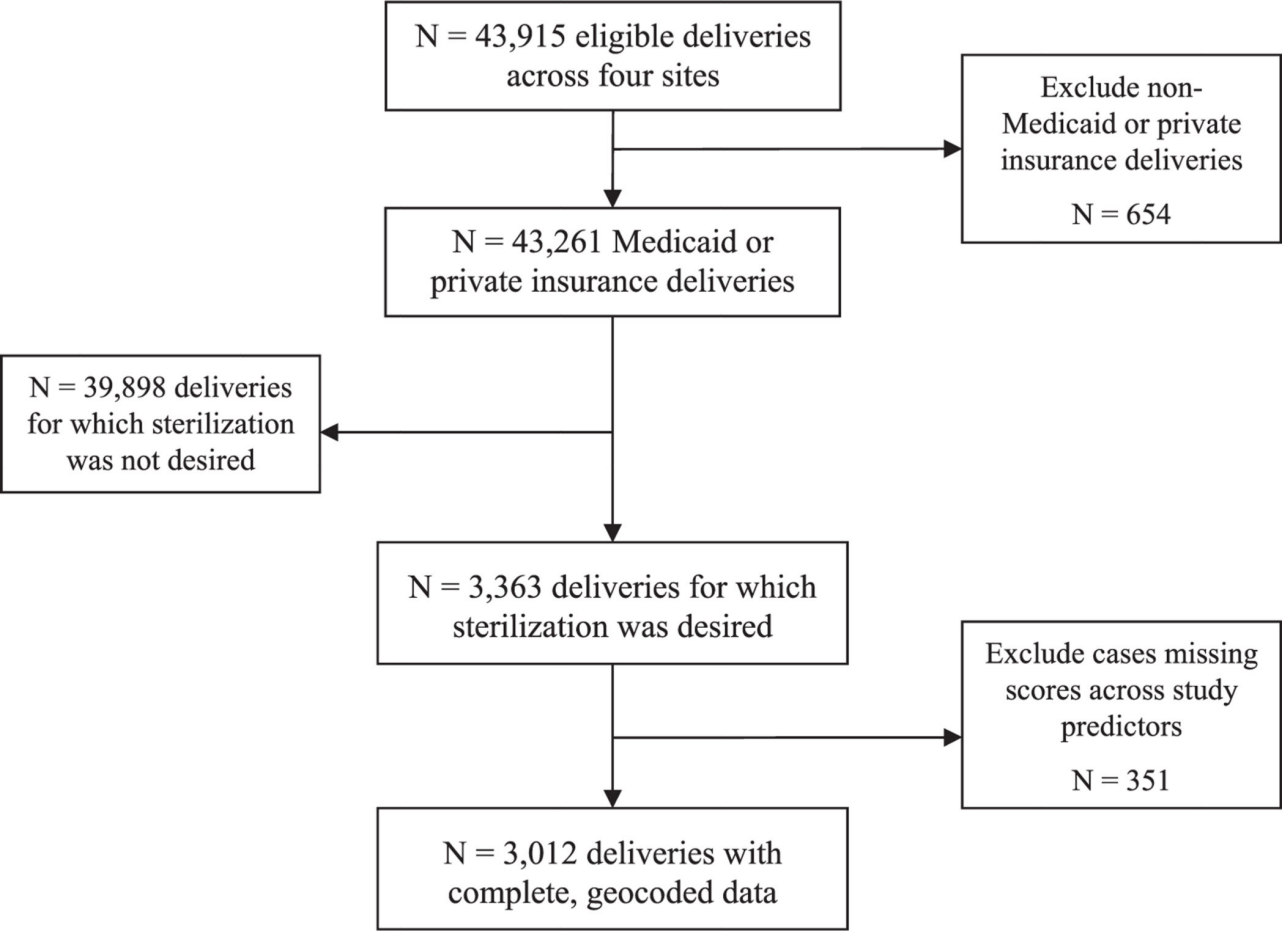
Flowchart of sample inclusion.

The final cohort included 3012 patients (Figure 1). Out of 43 915 eligible deliveries across all four sites, 98% of which were Medicaid or private insurance, sterilization was desired for 3363 (7.7%). Approximately 10% of the cohort had missing data across analysis variables; patients were most likely to have missing data on BMI (7.41%), ethnic identity (1.81%) and marital status (1.28%). The final sample included 3012 deliveries with complete data across study predictors (including ADI-3 scores generated from street-level matched addresses) (Figure 1). Of the final sample, 69% (*n* = 2075) of women had Medicaid insurance and 31% (*n* = 937) of women had private insurance. Across the sample, subdomains of the ADI-3 were moderately to strongly correlated in directions as expected (economic hardship with financial strength; *r* = −0.62; economic hardship with educational attainment; *r* = −0.31 and financial strength with educational attainment; *r* = 0.31).

Those who did not achieve permanent contraception at discharge (*n* = 1251), compared to those who did (*n* = 1761), lived in neighbourhoods with a greater mean economic hardship (117.20 vs. 109.75), less financial strength (88.74 vs. 94.27) and approximately the same educational attainment (97.50 vs. 97.58); differences were reflected in global ADI mean score differences (117.20 vs. 109.75) (Table 1). Compared to those who did achieve permanent contraception, those who did not achieve permanent contraception by discharge were more likely to have Medicaid insurance, have a Black and non-Hispanic racial and ethnic identity, were slightly younger with higher parity before delivery, had vaginal delivery, were unmarried and had slightly higher BMI.

Those with inadequate prenatal care (*n* = 1559), compared to those with adequate prenatal care (*n* = 1453), lived in neighbourhoods with greater mean financial strength (93.41 vs. 90.43) and economic hardship (116.31 vs. 114.7) and lower educational attainment (95.49 vs. 99.75) (Table 2). These neighbourhood differences were marginally reflected in differences across global ADI (113.30 vs. 112.52). Those with inadequate, compared to adequate, prenatal care were more likely to be non-Black, Hispanic, have higher parity before delivery, have slightly greater gestational age, were more likely to have delivered vaginally, and had lower BMI.

### Multivariate results

**FIGURE 2:**
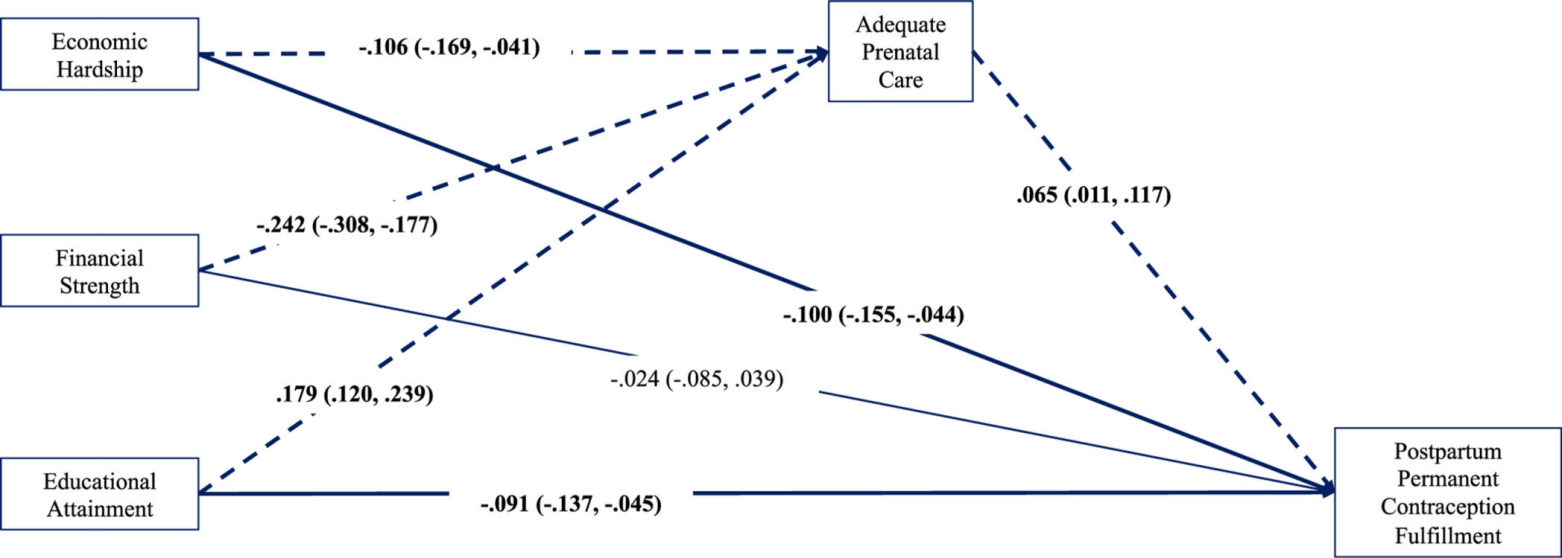
Observed path model for full sample (*N* = 3012). Standardized path coefficients are presented with 95% CIs. Bolded lines indicate direct effects. Dashed lines indicate indirect effects. The model adjusted for all covariates (insurance type, self-reported race and ethnicity, marital status, maternal age at delivery, parity up until delivery, weeks of gestation at delivery, delivery type and body mass index). When modelling global ADI, only relationships between ADI and postpartum permanent contraception fulfilment (*β* = −0.048, 95% CI: −0.098, −0.002) and adequacy of prenatal care and postpartum permanent contraception fulfilment (*β* = 0.057, 95% CI: 0.005, 0.109) emerged; no indirect effects were measured. ADI, Area Deprivation Index; CI, confidence interval.

Figure 2 illustrates the pathways from the ADI-3 to fulfilment of permanent contraception via adequacy of prenatal care for the full sample. Because the model was fully saturated with all observed variables and zero degrees of freedom, model fit is shown to be perfect^32^ with CFI = 1.00, RMSEA = 0.00 and SRMR = 0.000. The model explained 45.2% and 9.2% of the variance in permanent contraception fulfilment and achieving adequate prenatal care, respectively. After adjusting for covariates, having adequate prenatal care predicted a *z* score 0.065 standard deviations above the mean in the continuous latent response of achieving desired postpartum contraception fulfilment at discharge (*β* = 0.065, 95% CI: 0.011, 0.117).

**FIGURE 3:**
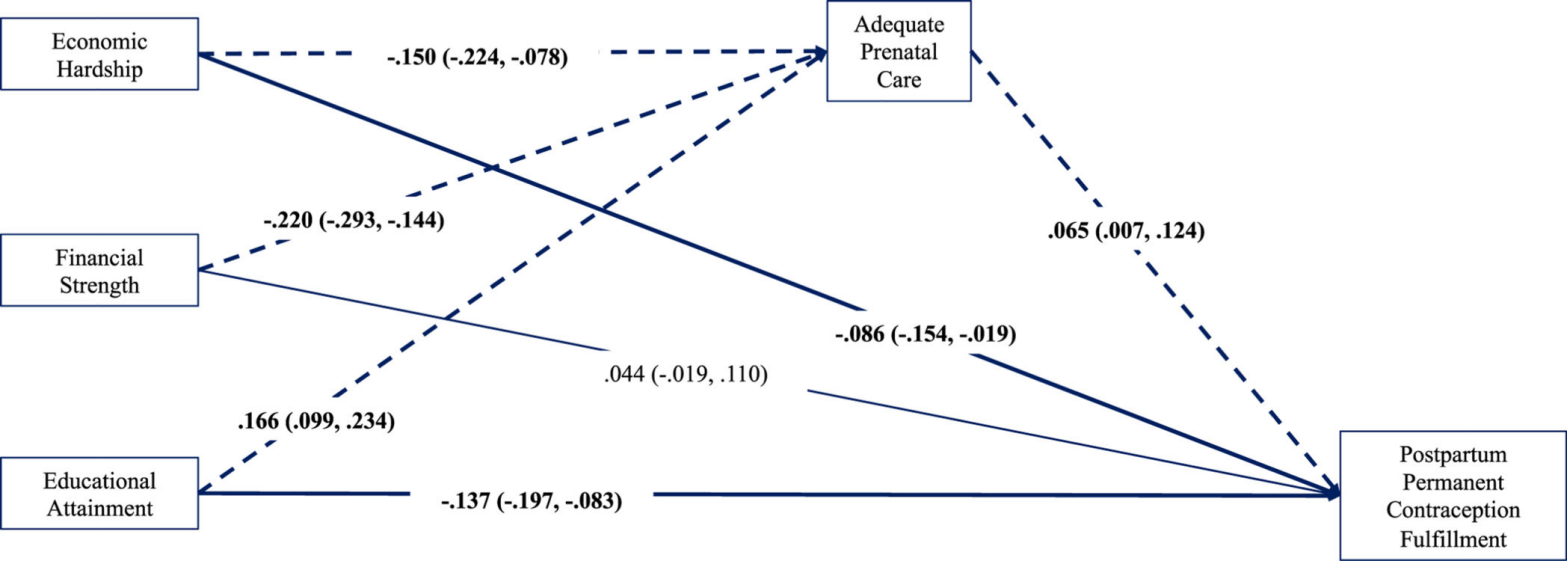
Observed path model for Medicaid patients (*n* = 2075). Standardized path coefficients are presented with 95% CIs. Bolded lines indicate direct effects. Dashed lines indicate indirect effects. Model adjusted for all covariates. CI, confidence interval.

**FIGURE 4:**
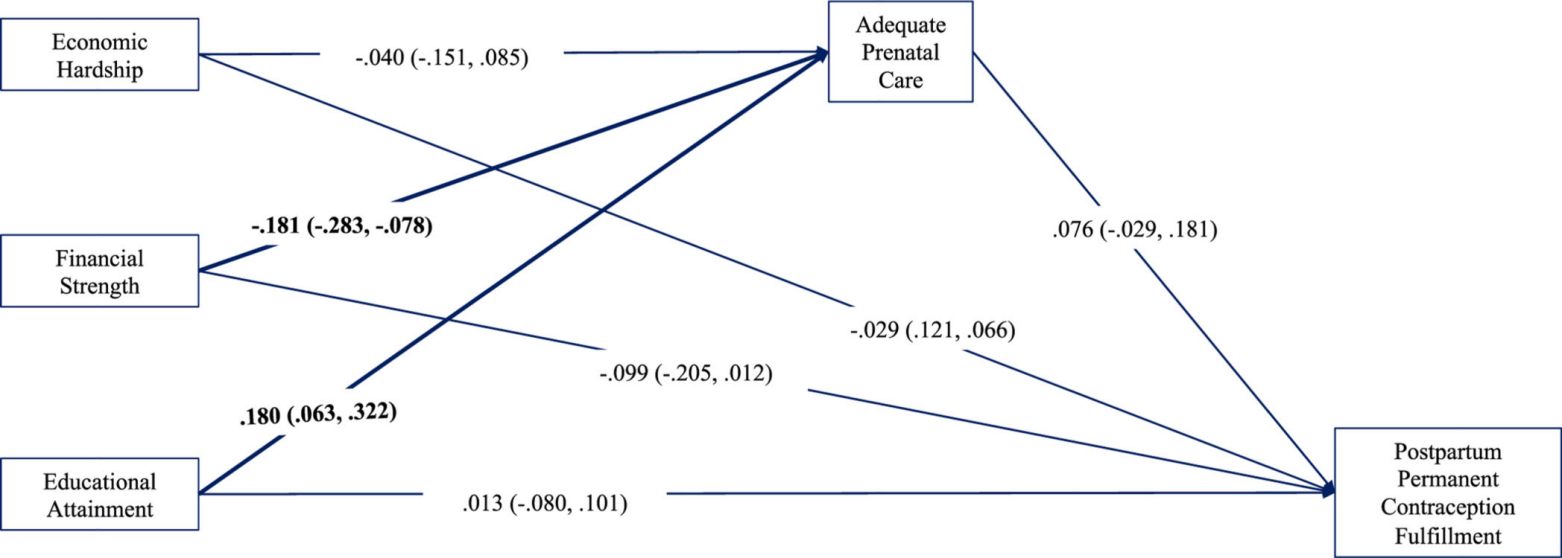
Observed path model for privately insured patients (*n* = 937). Standardized path coefficients are presented with 95% CIs. Bolded lines indicate significant direct effects. Model adjusted for all covariates. CI, confidence interval.

Results evidenced consistently small but measurable partial indirect (i.e., mediating) effects of neighbourhood socioeconomic conditions on permanent contraception fulfilment through adequacy of prenatal care in the full sample (Figure 2). Living in neighbourhoods with less economic hardship and inequality (indirect effect −0.007, 95% CI: −0.015, −0.001), less financial strength (indirect effect −0.016, 95% CI: −0.030, −0.002) and greater educational attainment (indirect effect 0.012, 95% CI: 0.002, 0.023) predicted having adequate prenatal care which, in turn, predicted achievement of permanent contraception by discharge. Similar small indirect effects were identified in the subsample of patients with Medicaid (Figure 3), where living in neighbourhoods with less economic hardship and inequality (indirect effect −0.010, 95% CI: −0.023, −0.001), less financial strength (indirect effect −0.014, 95% CI: −0.031, −0.002) and greater educational attainment (indirect effect 0.011, 95% CI: 0.001, 0.024) predicted having adequate prenatal care which, in turn, predicted achieving permanent contraception by discharge. For privately insured patients, adequacy of prenatal care conveyed no measurable effect of neighbourhood socioeconomic conditions on sterilization achievement (Figure 4).

Findings from moderation analysis indicate that the negative effect of neighbourhood financial strength on achieving permanent contraception was small but measurable for privately insured patients (*β* = −0.101, 95% CI: −0.193, −0.010) but not for patients with Medicaid insurance (supplemental table). Additionally, the negative effect of neighbourhood financial strength on achieving adequate prenatal care was over twice as strong among patients with Medicaid (*β* = −0.221, 95% CI: −0.293, −0.149). Finally, among patients with Medicaid, living in neighbourhoods of lower educational attainment predicted achievement of permanent contraception by discharge (*β* = −0.012, 95% CI: −0.017, −0.007) while no such relationship was detected for their privately insured counterparts.

## DISCUSSION

This multisite study investigated links between specific aspects of neighbourhood socioeconomic position (i.e., neighbourhood economic hardship, financial strength and educational attainment) and fulfilment of permanent contraception, both directly and indirectly through adequacy of prenatal care, and how insurance status moderates those relationships. We find that living in neighbourhoods with less economic hardship, less financial strength and greater educational attainment predicts achievement of adequate prenatal care which, in turn, predicts achievement of desired permanent contraception, and that some of these relationships are conditioned by insurance status. Though a small number of prior studies have examined associations between neighbourhood characteristics, adequacy of prenatal care and permanent contraception fulfilment, this study makes critical contributions for its examination of what, specifically, about neighbourhood socioeconomic position, and the opportunity structures it affords, may be relevant to these relationships.

One of our key findings suggests that adequacy of prenatal care may partially transmit the effects of neighbourhood economic hardship, financial strength and educational attainment on patients’ fulfilment of permanent contraception, and that these indirect effects may be accentuated among patients with Medicaid insurance. Our finding that having adequate prenatal care predicted the achievement of postpartum permanent contraception is consistent with that of prior studies.^12,13,35^ For patients with Medicaid insurance, these increased contacts with healthcare providers are particularly important given the federally mandated 30-day waiting period that must elapse between when a patient signs the Medicaid consent form, meant to formally document their noncoerced decision and when they can receive permanent contraception.

Our additional findings that patients living in neighbourhoods with less economic hardship and inequality, and in neighbourhoods with greater educational attainment, predicted achievement of adequate prenatal care aligns with other work emphasizing the role of neighbourhood environments in enhancing or constricting access to health and health-promoting resources in general,^36,37^ and reproductive health outcomes in particular.^10,38^ For example, a systematic review of factors predicting inadequate use of prenatal care in high-income countries has found that individuals residing in economically distressed neighbourhoods, which tend also to have lower levels of educational attainment, are more likely to have inadequate or late-entry prenatal care.^39^ Given the number and magnitude of daily social and economic stressors such neighbourhoods may impart, late entry into prenatal care may be one consequence of individuals having to negotiate other competing attentional or resource demands and barriers.^15,40^ This finding suggests that policies integrating health and social care interventions in the clinic for socioeconomically vulnerable patients (e.g., providing transportation assistance), as well as health care system-anchored investments in community development,^41^ may be germane in promoting access to prenatal care and reproductive health care equity.

We found an additional direct effect of neighbourhood economic hardship predicting patients’ lower likelihood of achieving postpartum permanent contraception. These results align with prior single-site research evidencing that living in more socioeconomically vulnerable neighbourhoods is associated with a greater likelihood of having Medicaid insurance.^10^ As such, the observed effects of neighbourhood economic hardship may reflect the potentially prohibitive barriers embedded within Medicaid policies for permanent contraception. Our finding that living in neighbourhoods with lower levels of educational attainment is directly associated with a higher likelihood of achieving permanent contraception is plausibly affected by the operationalization of this subdomain of the ADI-3. Namely, measurement work on the development of the ADI-3 underscores that the neighbourhood educational attainment subdimension, which includes the US census indicator for the percent of households in a neighbourhood with more than one person per room, at least partially reflects female fertility behaviour.^18^ Women with less education tend to have greater parity, and greater parity is known to predict higher odds of achieving desired permanent contraception.^13,42^ Given that having Medicaid insurance has been associated with having achieved lower levels of education,^43^ this likely also contextualizes our moderation finding where multigroup analyses revealed an inverse association between neighbourhood educational attainment and fulfilment of permanent contraception for patients with Medicaid insurance, but no such relationship was detected for those privately insured. This finding may also reflect bias among physicians to perform permanent contraception procedures among patients with higher parity because they have fewer concerns about those patients’ regret of the procedure.

Finally, our study reveals a somewhat paradoxical negative effect of neighbourhood financial strength; patients living in neighbourhoods with less financial strength are observed to have a higher probability of achieving adequate prenatal care. While other interpretations are possible, we contend that this finding reflects a nuance of the Kotelchuck Index,^17^ the measure by which we operationalized adequacy of prenatal care. Kotelchuck Index scores are generated based on two primary components: when prenatal care is initiated during pregnancy, and the total number of prenatal care visits as a proportion of the number recommended by the American College of Obstetricians and Gynecologists assuming uncomplicated pregnancies.^17^ However, women with high-risk pregnancies are likely to be monitored more closely during the prenatal period and attend more frequent prenatal visits. As such, for some women, scores that reflect greater adequacy of prenatal care may more specifically indicate numerous prenatal visits as a marker of higher-risk pregnancies. Further, research suggests that women with Medicaid are at higher risk of having low-birthweight infants or preterm births compared to their privately insured counterparts,^44^ outcomes that have also been associated with neighbourhood disadvantage.^45,46^

We thus interpret these dynamics as reflected in our moderation finding that, for women with Medicaid but not private insurance, living in neighbourhoods with less financial strength is associated with a higher likelihood of having adequate prenatal care. A somewhat similar phenomenon may explain other moderation findings that living in neighbourhoods with less financial strength predicts a higher probability of achieving permanent contraception by discharge but only among private patients. When holding all else equal between Medicaid and privately insured patients—for instance, given equal numbers of prenatal care visits to voice preferences for permanent contraception—it may be that women on private insurance living in financially weaker neighbourhoods are more likely to achieve permanent contraception because they do not have to contend with the mandated consent and waiting period that women with Medicaid do. Thus, ongoing policy research should continually re-examine the federally mandated Medicaid consent form and waiting period to best support women’s reproductive autonomy.

Study findings should be considered in light of limitations. First, given the retrospective cohort design, our study is constrained by data limitations associated with the electronic medical record (e.g., lack of comprehensive and standardized measurement of racial and ethnic identity).^47^ Second, administrative neighbourhood data such as those from the US census are imperfect abstractions of patients’ community environments and may not best account for the role of neighbourhood phenomena in patients’ everyday lives and reproductive healthcare. Similarly, inferences made about individual-level experiences based on neighbourhood data are vulnerable to ecological fallacies wherein patients in our sample may not reflect the same socioeconomic position as their neighbourhood populations. Additionally, the cross-sectional nature of the study precludes insight into the dynamic nature across time between prenatal care or permanent contraception fulfilment and neighbourhood residence and neighbourhood change. Future longitudinal research is needed to test these links as well as further examine the endogeneity of study variables. Also, while the majority (93%) of neighbourhoods in our study included five or fewer patients, our modelling did not account for any shared variance among patients in the same or bordering neighbourhoods (i.e., our models did not account for potential clustering within neighbourhood). Finally, given our small indirect effect sizes, our multivariate modelling could not account for the full breadth of social, economic and health circumstances that are consequential in determining patients’ adequacy of prenatal care or fulfilment of desired permanent contraception. It is likely that current data do not fully capture all the ways in which the lives of women with Medicaid insurance differ from those with private insurance. Future inquiry that employs quantitative approaches to deductively test additional hypotheses and leverages qualitative data to inductively build a theory, of mechanisms linking neighbourhood conditions and permanent contraception fulfilment is warranted.

## CONCLUSION

Our study offers multiple substantive and methodological contributions to the literature. First, its multisite design, as well as our utilization of US-referenced neighbourhood socioeconomic position scores, enhances the generalizability of findings to other geographic regions and healthcare systems. Second, this is the first study to model the ADI-3 in examining the nuance and variability of health outcomes and mechanisms according to precise and empirically defined domains of neighbourhood socioeconomic position. Third, study findings illuminate potential areas for refining the measurement of prenatal care adequacy given confounding relationships between individual and place-based socioeconomic vulnerability, high-risk pregnancy and frequency of prenatal care visits. Finally, our study findings suggest that future inquiry and policy might examine the structural, place-based characteristics of women’s lives—in addition to person-, provider-, or systems-level factors—that matter to their reproductive health needs and goals. Clarifying the relationships between broader structures of social and economic opportunity that shape where women live and how they access reproductive healthcare is critical to promoting reproductive autonomy and healthcare equity.

## Supplementary Material

Supinfo

## Figures and Tables

**TABLE 1:** Characteristics by permanent contraception achievement at discharge for patients who desired postpartum permanent contraception across all study sites (*N* = 3012).

	No permanent contraception at discharge, *n* = 1251	Permanent contraception at discharge, *n* = 1761
Medicaid (%)	978 (78.2)	1097 (62.3)
ADI	117.20 (115.98, 118.42)	109.75 (108.63, 110.84)
ADI-3: Neighbourhood economic hardship and inequality	120.74 (119.23, 122.25)	111.83 (110.60, 113.06)
ADI-3: Neighbourhood financial strength	88.74 (87.78, 89.70)	94.27 (93.29, 95.25)
ADI-3: Neighbourhood educational attainment	97.50 (96.68, 98.32)	97.58 (96.74, 98.41)
Maternal age	31.33 (31.05, 31.61)	32.71 (32.46, 32.96)
Parity less than 2 at admission	310 (24.8)	505 (28.7)
Gestational age at delivery in weeks	37.48 (37.31, 37.66)	37.64 (37.52, 37.75)
Vaginal delivery (%)	1049 (83.9)	507 (28.8)
Maternal race (%) Black	602 (48.1)	640 (36.3)
White	384 (30.7)	659 (37.4)
Asian	36 (2.9)	53 (3.0)
Declined or unknown	135 (10.8)	262 (14.9)
Other	94 (7.5)	147 (8.3)
Maternal ethnicity non-Hispanic (%)	952 (76.1)	1282 (72.8)
Married (%)	335 (26.8)	791 (44.9)
BMI at delivery (kg/m^2^)	35.74 (35.29, 36.20)	34.67 (34.31, 35.03)

*Note*: Presented as *n* (%) or mean (95% CI).

Abbreviations: ADI, area deprivation index; BMI, body mass index; CI, confidence interval.

**TABLE 2:** Characteristics by adequacy of prenatal care for patients who desired postpartum sterilization across all study sites (*N* = 3012).

	Inadequate prenatal care, *n* = 1559	Adequate prenatal care, *n* = 1453
Medicaid (%)	1071 (68.70)	1004 (69.10)
ADI	113.3 (111.94, 114.32)	112.52 (111.37, 113.67)
ADI-3: Neighbourhood economic hardship and inequality	116.31 (114.93, 117.69)	114.7 (113.35, 116.05)
ADI-3: Neighbourhood financial strength	93.41 (92.35, 94.47)	90.43 (89.51, 91.35)
ADI-3: Neighbourhood educational attainment	95.49 (94.60, 96.38)	99.75 (98.98, 100.51)
Maternal age	32.04 (31.77, 32.30)	32.25 (31.98, 32.52)
Parity less than 2 at admission (%)	379 (24.3)	436 (30.0)
Gestational age in weeks	37.73 (37.59, 37.87)	37.41 (37.26, 37.55)
Vaginal delivery (%)	845 (54.2)	711 (48.9)
Maternal race (%) Black	596 (38.2)	646 (44.5)
White	544 (34.9)	499 (34.3)
Asian	50 (3.2)	39 (2.7)
Declined or unknown	257 (16.5)	140 (9.6)
Other	112 (7.2)	129 (8.9)
Maternal ethnicity non-Hispanic (%)	1128 (72.4)	1106 (76.1)
Married (%)	570 (36.6)	556 (38.3)
BMI at delivery (kg/m^2^)	34.49 (34.09, 34.88)	35.79 (35.38, 36.20)

*Note*: Presented as *n* (%) or mean (95% CI).

Abbreviations: ADI, area deprivation index; BMI, body mass index; CI, confidence interval.

## Data Availability

Research data are not shared given privacy or ethical restrictions.
