# Supplementary material for: Neighbourhood socioeconomic position, prenatal care and fulfilment of postpartum permanent contraception: Findings from a multisite cohort study
Source: Reprod Female Child Health. Author manuscript; Available in PMC 2025 Mar 1. (PMC11087039; doi:10.1002/rfc2.64)
Supplement: Supinfo [file NIHMS1938833-supplement-Supinfo.docx]

**Supplemental Table.** Testing for Equivalence of Path Analysis Model Across Medicaid and Privately Insured Patients

| **Model** | | **Description** | **RMSEA** | **(90% CI)** | **SRMR** | **CFI** | **Ref** | **ΔRMSEA** | **ΔCFI** |
| --- | --- | --- | --- | --- | --- | --- | --- | --- | --- |
|  | | Medicaid | 0.000 | (0.000 - 0.000) | 0.000 | 1.000 |  |  |  |
|  | | Private | 0.000 | (0.000 - 0.000) | 0.000 | 1.000 |  |  |  |
| 1 | | Configural (Unconstrained) Model. | 0.000 | (0.000 - 0.000) | 0.000 | 1.000 |  |  |  |
| 2 | | All paths invariant. | 0.027 | (0.018 - 0.037) | 0.112 | 0.968 | 1 | 0.027 | -0.032 |
| 3 | | Paths between adequacy of prenatal care and neighborhood financial strength free to vary. | 0.024 | (0.014 - 0.034) | 0.084 | 0.975 | 1 | 0.024 | -0.025 |
| 4 | | Paths between adequacy of prenatal care and neighborhood financial strength; sterilization and neighborhood financial strength free to vary. | 0.022 | (0.011 - 0.033) | 0.101 | 0.980 | 1 | 0.022 | -0.020 |
| 5 | | Paths between adequacy of prenatal care and neighborhood financial strength; sterilization and neighborhood financial strength; adequacy of prenatal care and ‘other’ race free to vary. | 0.017 | (0.000 - 0.028) | 0.102 | 0.989 | 1 | 0.017 | -0.011 |
| 6 | | Paths between adequacy of prenatal care and neighborhood financial strength; sterilization and neighborhood financial strength; adequacy of prenatal care and ‘other’ race; sterilization and neighborhood educational attainment free to vary. | 0.011 | (0.000 - 0.024) | 0.075 | 0.995 | 1 | 0.011 | -0.005 |
|  | |  |  |  |  |  |  |  |  |
|  |  |  |  |  |  |  |  |  |  |
